# A systematic review and meta‐analysis on the safety and efficacy of Aquablation

**DOI:** 10.1002/bco2.70292

**Published:** 2026-10-07

**Authors:** Kimberley Chan, Nico Christian Grossmann, Matthew Feyissa, Jeremy Nettleton, Philip Charlesworth, Neil Barber, Jeremy Teoh, Ben Challacombe, Garrett Pohlman, Evangelos Liatsikos, Dean Elterman, Nikhil Vasdev

**Affiliations:** ^1^ Hertfordshire and Bedfordshire Urological Cancer Centre, Department of Urology Lister Hospital, East and North Hertfordshire NHS Teaching Trust Stevenage UK; ^2^ Clinic for Urology University Teaching and Research Hospital of the University of Lucerne Lucerne Switzerland; ^3^ Cheltenham General Hospital, Gloucestershire Hospitals NHS Foundation Trust Cheltenham UK; ^4^ The Royal Marsden NHS Trust London UK; ^5^ Department of Urology Frimley Park Hospital London UK; ^6^ Faculty of Medicine The Chinese University of Hong Kong Hong Kong; ^7^ Department of Urology Medical University of Vienna Vienna Austria; ^8^ Guy's and St Thomas' NHS Foundation Trust London UK; ^9^ Kearney Urology Center PC Kearney Nebraska USA; ^10^ Department of Urology University of Patras Patras Greece; ^11^ Division of Urology, Department of Surgery University Health Network, University of Toronto Toronto ON Canada; ^12^ School of Health, Medicine and Life Sciences University of Hertfordshire Hatfield UK

**Keywords:** Aquablation, benign prostate hyperplasia, prostate, waterjet

## Abstract

**Objectives:**

This work aimed to evaluate the efficacy and safety of Aquablation for the treatment of benign prostatic enlargement.

**Methods:**

A systematic review was conducted in accordance with PRISMA 2020 guidelines (PROSPERO CRD420261401865). A comprehensive PubMed search identified prospective studies and randomised controlled trials published up to January 2026. Studies including ≥30 patients reporting clinical outcomes of Aquablation were included. Guidelines, case reports, review articles, letters, retrospective studies with pooled data were excluded. Primary outcomes were International Prostate Symptom Score, maximum flow rate, postvoid residual and sexual function. Secondary outcomes included perioperative parameters and complications. Random‐effects meta‐analysis using DerSimonian–Laird was performed for outcomes reported in three or more studies.

**Results:**

Fifteen studies comprising 1580 patients were included. Mean age and prostate volume were 67.0 years and 82.1 cc, respectively. Aquablation significantly improved urinary outcomes, with a pooled IPSS reduction of −15.80 at 6 months (95% confidence interval [CI] −17.88 to −13.73; *I* ^2^ = 97.8%), sustained to 36 months. *Q*_max_ increased by 11.0 mL/s (95% CI 8.93–13.02; *I* ^2^ = 95.1%), while PVR decreased by 103.8 mL (95% CI −139.5 to −68.0; *I* ^2^ = 98.1%) at 6 months. Ejaculatory dysfunction occurred in 7.4% of patients, with no reported erectile dysfunction. The transfusion rate was 2.5%, with lower rates observed using focal bladder neck cautery. Most complications were low grade.

**Conclusion:**

Aquablation provides durable and significant improvements in urinary outcomes with a favourable safety profile and low rates of sexual dysfunction, supporting its role as an effective surgical option for BPE.

## 1 INTRODUCTION

Benign prostatic hyperplasia (BPH) is a common age‐related benign urological condition. Its histological prevalence increases from approximately 8% in men in their 40s to 50% in their 60s and 80% in their 80s.^1^ BPH is characterised by hyperplasia of stromal and glandular tissue within the prostate transition zone, which may cause bladder outlet obstruction and lower urinary tract symptoms (LUTS), adversely affecting quality of life (QoL).^2^ Untreated obstruction may result in complications such as acute urinary retention and renal impairment.^3^ Initial management includes watchful waiting and lifestyle modification. Moderate‐to‐severe LUTS may be treated with α1‐blockers, which reduce prostatic and bladder neck smooth muscle tone, while 5‐alpha reductase inhibitors reduce prostate volume in men with enlarged prostates.^4^, ^5^ However, approximately 26% of patients discontinue pharmacological treatment within 2 years because of adverse effects or inadequate symptom control, with some subsequently requiring surgical intervention.^6^

Transurethral resection of prostate (TURP) has historically been considered the reference technique for surgical management of LUTS. Although TURP is very effective at treating LUTS, it is associated with undesirable sexual side effects such as retrograde ejaculation and ejaculatory disorder, both observed in approximately 65%.^7^ A survey on 300 patients having undergone or considering BPH surgery considered maintenance of erectile (95%) and ejaculatory function (92%) as an important preoperative consideration regardless of age.^8^ Other treatment options are therefore being developed to challenge TURP, as preservation of sexual function has become increasingly recognised as important. More recently, Aquablation (Aquabeam or HYDROS Robotic System, Procept BioRobotics, San Jose, CA, USA) has emerged as a novel waterjet resection therapy. First described in 2015, Aquablation combines imaging guidance for surgical planning and mapping and robotics with high velocity heat free saline for targeted removal of prostatic tissue.^9^ Both the Aquabeam and the HYDROS Robotics Systems consist of a tower, handpiece and transrectal ultrasound probe (TRUS). Under TRUS guidance, the 24F handpiece is inserted into the prostatic urethra and secured using a bed‐mounted articulating arm. The surgeon plans the resection which is subsequently robotically executed using a high‐velocity waterjet to resect adenomatous tissue while avoiding the verumontanum and ejaculatory ducts. The treatment remains within the surgical capsule. After performing the treatment passes to optimise the resection, the handpiece is removed. Haemostasis is achieved using focal bladder neck cautery technique.^10^ A three‐way urinary catheter is inserted at the end of the procedure.

An increasing number of studies have evaluated the functional and sexual outcomes of Aquablation over the past decade, with substantial evidence emerging in recent years. Previous systematic reviews have demonstrated significant improvements in urinary symptoms and flow rates^10^, ^11^ and favourable preservation of sexual and ejaculatory function.^12^ However, the existing reviews have primarily focused on specific outcomes, and an up‐to‐date synthesis of the overall safety and efficacy of Aquablation remains limited. This systematic review therefore aims to evaluate the contemporary evidence of the safety and efficacy of Aquablation, with particular consideration of functional, sexual and perioperative outcomes.

## 2 METHODS

### 2.1 Search strategy and selection criteria

The systematic review was conducted in accordance with Cochrane Collaboration Preferred Reporting Items for Systematic reviews and Meta‐Analyses (PRISMA) 2020 statement (PROSPERO CRD420261401865).^13^ A comprehensive literature search was conducted using PubMed, including all studies published up to January 2026. The search strategy included ‘Aquablation’, ‘Waterjet resection’, ‘Robotic surgery’ and ‘Benign prostate hyperplasia’. Titles and abstracts of articles identified by search strategy keywords were retrieved for screening. Our selection criteria include (a) studies with human subjects, (b) randomised controlled trials or prospective studies, (c) English‐language studies and (d) studies that reported Aquablation outcomes. For cohorts published multiple times, only the most recent publications were included. Guidelines, case reports, review articles, letters, retrospective studies and studies with pooled data or less than 30 patients were excluded. Title and abstract screening were performed by two independent evaluators against study selection criteria. Any discrepancies were resolved. After screening, full text from the remaining articles were retrieved and assessed, with articles not meeting selection criteria excluded. Consensus on included articles was achieved by third evaluator, after review of full text from articles against selection criteria. Figure 1 shows the flow diagram detailing the search strategy, screening and article selection for evidence synthesis.

**FIGURE 1:**
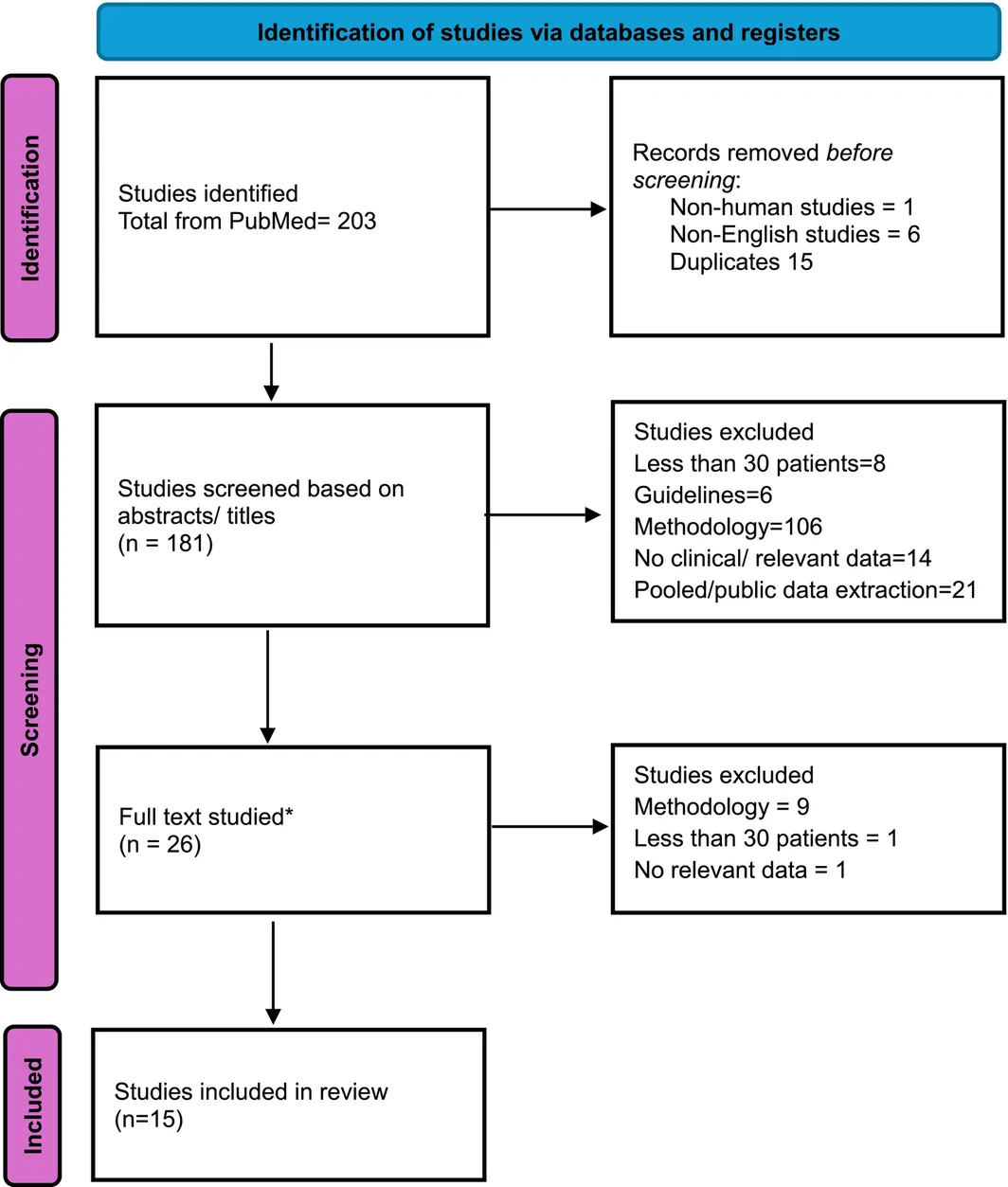
PRISMA flow diagram detailing the search strategy and identification of studies used in evidence synthesis.

### 2.2 Study aims and data extraction

Data on baseline characteristics such as age, prostate size, median lobe obstruction and clinical urinary parameters were extracted from eligible studies when provided. Further data were extracted for our primary and secondary outcomes. The primary objective was to assess the efficacy of Aquablation in patients with symptomatic benign prostate enlargement in terms of clinical and functional outcomes. Primary outcome measures include International Prostate Symptom Score (IPSS), maximum urinary flow (*Q*_max_), postvoid residual (PVR), Male Sexual Health Questionnaire (MSHQ) and International Index of Erectile Function (IIEF‐5). The secondary objective was to evaluate the safety of Aquablation. Secondary outcome measures include post‐operative haemoglobin changes, Clavien–Dindo events, operative time and return to theatre.

### 2.3 Statistical analysis

Data were inputted into a structured excel sheet and reviewed with any missing data identified and imputed. Algebraic recalculation was performed using within‐study summary data, including estimation of means from medians, standard deviations (SDs) from interquartile ranges and SDs from minimum–maximum ranges according to methodological recommendations.^14^ If algebraic recalculation was not possible, missing SDs were imputed using a prognostic (sample‐based) approach, using information from the entire pooled sample.^15^ Following imputation, the data were transformed to derive outcome‐specific effect estimates. Between‐study variance was estimated using DerSimonian–Laird with statistical heterogeneity summarised using *τ* and *I* ^2^. Random effects meta‐analysis was conducted on outcomes reported by at least three studies, and outcomes reported by fewer than three studies were included for descriptive and discussion purposes only.

## 3 EVIDENCE SYNTHESIS

### 3.1 Search strategy

The literature search identified 203 papers. Fifteen duplicates, one nonhuman study and six non‐English studies were automatically excluded leaving 181 papers for screening by title and abstract. Based on our selection and exclusion criteria, a further 155 papers were excluded. The reasons were as follows: methodology (106), articles with pooled/public data extraction (21), articles without clinical data (14), articles with less than 30 patients (8) and guidelines (6). After full text evaluation, a further 11 papers were excluded based on methodology (9), articles with less than 30 patients (1) and articles without clinical data (1). The remaining 15 articles were included in the systematic review (Tables 1 and 2).

**TABLE 1:** Included studies with patient characteristics and baseline functional outcomes.

					Patient characteristics		Baseline functional outcomes			
Study	Study type	Country	*n*	Treatment year	Age, years	Prostate volume, mL	IPSS	QoL	*Q*_max_, mL/s	PVR, mL
Desai et al., 2018	Single Arm	India	47	2014–2015	66.0 (6.0)	48.0 (24.0)	24.4 (4.4)	4.5 (0.9)	7.1 (4.1)	119.0 (122.0)
Gilling et al., 2022	RCT	Multinational	116	2015–2016	66.0 (7.3)	54.1 (16.2)	22.9 (6.0)	4.8 (1.1)	9.4 (3.0)	97.0 (179.0)
Misrai et al., 2019	Single Arm	France	30	2017	68.0 (10.4)	60.0 (17.8)	18.5 (6.7)	5.0 (1.5)	NR	NR
Bhojani et al., 2023	Single Arm	USA and Canada	101	2017	68.0 (6.6)	107.0 (20.2)	23.2 (6.3)	4.6 (1.3)	8.7 (3.4)	131.0 (125.0)
Bach et al., 2019	Cohort	Germany	118	2017–2018	69.0 (8.0)	64.0 (32.0)	21.1 (6.8)	4.6 (1.3)	10.8 (5.8)	158.9 (282.9)
Bach et al., 2020	Single Arm	Multinational	178	2017–2018	68.0 (8.5)	59.0 (26.9)	21.7 (7.1)	4.6 (1.2)	9.9 (5.3)	108.0 (108.0)
Kasraeian et al., 2020	Cohort	USA	55	2018–2019	67.0 (8.2)	100.0 (44.0)	21.6 (6.9)	4.3 (1.1)	7.4 (3.9)	NR
Labban et al., 2021	Cohort	Lebanon	59	2018–2020	68.0 (7.9)	71.4 (31.3)	19.9 (6.2)	4.1 (1.5)	NR	228.0 (48.0)
Amparore et al., 2024	Single Arm	USA	109	2018–2023	66.0 (7.4)	62.0 (19.4)	22.0 (6.7)	5.0 (0.7)	8.4 (2.8)	83.3 (67.8)
Omidele et al., 2024	Cohort	USA	330	2019–2023	65.0 (7.3)	110.0 (48.7)	23.8 (8.4)	NR	6.4 (4.2)	121.8 (39.7)
Ritter et al., 2025	RCT	UK and Germany	98	2020–2024	65.2 (6.8)	105.3 (20.0)	20.7 (5.7)	4.2 (1.5)	10.7 (4.5)	101.2 (81.8)
Justo Quintas et al., 2025	NRCT	Spain	75	2021–2023	66.7 (8.2)	71.9 (34.8)	20.2 (7.1)	4.3 (1.4)	9.1 (3.6)	69.8 (48.5)
Hinata et al., 2025	Single‐Arm	Japan	103	2024	71.0 (7.0)	82.0 (24.4)	18.1 (9.0)	4.9 (1.3)	8.3 (4.4)	85.6 (107.2)
Zorn et al., 2025	Cohort	Canada	60	2024	69.0 (4.3)	115.0 (38.2)	29.7 (3.0)	5.2 (0.5)	5.9 (1.3)	408.0 (202.7)
Marhamati et al., 2025	Cohort	USA	101	2024–2025	66.9 (7.1)	66.3 (21.9)	22.0 (6.9)	4.4 (1.2)	10.6 (5.0)	161.0 (143.8)

**TABLE 2:** Procedure characteristics, functional and sexual outcomes and procedure complication rates.

			Procedure characteristics			Longest follow‐up functional outcome				Sexual health risk		SUI risk	Procedure complications						
Study	*N*	Prostate volume, mL	Operative time, min	Length of stay, days	Follow‐up, months	IPSS (longest)	QoL (longest)	*Q*_max_, mL/s (longest)	PVR, mL (longest)	ED	EjD	Stress incontinence	Haemostasis technique	Transfusion	Take‐back to theatre	CD1	CD2	CD3	CD4
Desai et al., 2018	47	48.0 (24.0)	35.0 (19.0)	3.10 (1.60)	3	5.0 (4.4)	0.3 (1.2)	16.5 (10.8)	43.0 (33.5)	0 (0.0%)	0 (0.0%)	0 (0.0%)	Mixed	1 (2.1%)	3 (6.4%)	6.4%	4.3%	10.6%	0.0%
Gilling et al., 2022	116	54.1 (16.2)	40.0 (14.9)	1.40 (0.69)	60	7.0 (6.3)	1.6 (NR)	17.3 (7.0)	42.2 (62.9)	0 (0.0%)	8 (6.9%)	0 (0.0%)	Mixed	1 (0.9%)	0 (0.0%)	33.6%	16.4%	6.0%	0.9%
Misrai et al., 2019	30	60.0 (17.8)	56.0 (10.4)	2.00 (0.69)	12	3.0 (3.7)	NR	NR	NR	0 (0.0%)	8 (26.7%)	0 (0.0%)	Traction	1 (3.3%)	1 (3.3%)	50.0%	13.3%	13.3%	0.0%
Bhojani et al., 2023	101	107.0 (20.2)	55.0 (14.3)	1.60 (0.90)	60	6.8 (4.6)	1.3 (1.3)	17.1 (9.8)	64.0 (64.0)	0 (0.0%)	15 (14.9%)	1 (1.0%)	Traction	10 (9.9%)	5 (5.0%)	18.0%	21.8%	13.9%	5.0%
Bach et al., 2019	118	64.0 (32.0)	20.0 (7.9)	NR	3	7.2 (5.2)	1.5 (1.3)	21.6 (12.8)	13.2 (19.4)	NR	0 (0.0%)	NR	Mixed	3 (2.5%)	4 (3.4%)	NR	8.5%	3.4%	0.0%
Bach et al., 2020	178	59.0 (26.9)	24.0 (14.9)	2.20 (0.69)	12	6.5 (4.8)	1.4 (1.4)	20.8 (11.2)	61.0 (74.0)	0 (0.0%)	14 (7.9%)	0 (0.0%)	Mixed	5 (2.8%)	14 (7.9%)	20.0%	9.0%	13.0%	0.0%
Kasraeian et al., 2020	55	100.0 (44.0)	59.0 (14.9)	1.80 (0.69)	12	5.0 (4.0)	1.0 (1.4)	20.6 (10.8)	NR	0 (0.0%)	NR	NR	NR	1 (1.8%)	NR	NR	NR	NR	NR
Labban et al., 2021	59	71.4 (31.3)	48.0 (25.0)	2.20 (0.10)	12	7.5 (4.0)	1.4 (1.4)	NR	42.0 (33.5)	NR	10 (16.9%)	NR	Mixed	0 (0.0%)	3 (5.1%)	35.7%	35.7%	21.4%	7.1%
Amparore et al., 2024	109	62.0 (19.4)	52.0 (14.4)	3.60 (1.20)	12	3.0 (2.2)	1.0 (1.5)	17.1 (7.8)	20.7 (11.8)	0 (0.0%)	4 (3.7%)	0 (0.0%)	FBNC	3 (2.8%)	2 (1.8%)	7.2%	12.7%	1.8%	0.0%
Omidele et al., 2024	330	110.0 (48.7)	45.0 (14.9)	NR	48	6.9 (2.9)	NR	17.4 (5.5)	31.4 (11.5)	0 (0.0%)	1 (0.3%)	NR	NR	11 (3.3%)	13 (3.9%)	NR	NR	NR	NR
Ritter et al., 2025	98	105.3 (20.0)	58.1 (19.6)	NR	3	7.9 (5.1)	NR	19.8 (10.6)	48.0 (66.0)	0 (0.0%)	8 (8.2%)	0 (0.0%)	FBNC	0 (0.0%)	5 (5.1%)	NR	NR	8.2%	0.0%
Justo Quintas et al., 2025	75	71.9 (34.8)	49.1 (15.0)	1.63 (0.80)	6	5.0 (4.9)	1.3 (1.6)	20.3 (22.6)	20.3 (22.6)	0 (0.0%)	5 (6.7%)	0 (0.0%)	FBNC	1 (1.3%)	NR	6.6%	4.0%	0.0%	0.0%
Hinata et al., 2025	103	82.0 (24.4)	61.0 (21.6)	7.00 (4.30)	6	6.1 (5.0)	1.8 (1.3)	15.5 (7.8)	43.3 (6.0)	0 (0.0%)	0 (0.0%)	0 (0.0%)	FBNC	1 (1.0%)	0 (0.0%)	1.0%	5.8%	3.9%	0.0%
Zorn et al., 2025	60	115.0 (38.2)	65.0 (12.0)	0.20 (0.02)	1	6.6 (1.3)	0.9 (0.3)	26.3 (6.0)	39.0 (20.0)	0 (0.0%)	9 (15.0%)	2 (3.3%)	FBNC	0 (0.0%)	0 (0.0%)	NR	NR	NR	NR
Marhamati et al., 2025	101	66.3 (21.9)	50.7 (19.1)	0.06 (0.04)	3	9.2 (6.8)	1.9 (1.8)	18.3 (7.6)	67.3 (108.4)	NR	NR	0 (0.0%)	FBNC	0 (0.0%)	1 (1.0%)	4.0%	0.0%	1.0%	0.0%

### 3.2 Study characteristics

The 15 articles included 1580 patients treated with Aquablation. The population studied was European in three studies,^16^, ^17^, ^18^ American in four studies,^19^, ^20^, ^21^, ^22^ Canadian in one study,^23^ Middle Eastern in one study,^24^ Japanese in one study,^25^ Indian in one study^26^ and multicentric in five studies.^27^, ^28^, ^29^, ^30^

### 3.3 Baseline patient and perioperative characteristics

Across 15 studies including 1580 patients, the mean age of individuals undergoing Aquablation was 67.0 years ± 7.6. The mean prostate volume treated was 82.1 cc ± 39.7. Baseline functional outcomes demonstrated a mean IPSS of 22.2 ± 7.4. Among the included studies, 12 reported baseline QoL with a mean score of 4.6 ± 1.2. Additionally, 13 studies reported *Q*_max_, which averaged 8.6 mL/s ± 4.6, while the mean PVR was 130.8 mL ± 145. The mean follow‐up duration across studies was 22.4 months. The average operative time for Aquablation was 47.1 min ± 17.3. Among the 12 of 15 studies that reported hospital length of stay, the mean duration was 1.86 days ± 1.28.

### 3.4 Urinary outcomes

Pre and post treatment IPSS was only recorded in 14 of 15 studies at 6 months, 12 of 15 studies at 12 months, three of 15 studies at 24, 36 and 48 months and two studies at 60 months. Meta‐analysis showed significant and durable improvement in IPSS at evaluated follow up period of 48 months. At 6 months (*N* = 1250), pooled mean reduction in IPSS was −15.80 (95% CI −17.88 to −13.73; *I* ^2^ = 97.8%, *p* < 0.001). This improvement is sustained at 12 months (−15.69, 95% CI −17.01 to −14.38, *I* ^2^ = 90.2%, *p* < 0.001), through to longer follow‐up intervals at 24 months (−15.98, 95% CI −17.44 to −14.51, *I* ^2^ = 84.2%, *p* < 0.001) and 36 months (−15.97, 95% CI −17.00 to −14.94, *I* ^2^ = 68.5%, *p* < 0.001) (Table 3 and Figure 2). Similarly, of the 12 of 15 studies which reported QoL changes up till 6 months (*k* = 12, *N* = 1122), there is a decline in QoL score by −3.31 (95% CI −3.69 to −3.95, *I* ^2^ = 97.0%, *p* < 0.001) (Figure 3). This change is sustained through to 12 months. The interpretation of results beyond that is limited by lack of heterogeneity as many studies did not report QoL data beyond 12 months (*k* < 3), although studies which did report QoL score show a continued decline in QoL score at 36 months.

**TABLE 3:** Summary of pooled effect sizes for IPSS, QoL, *Q*_max_ and PVR.

Outcome	Interval	*k*	*N*	Estimate (95% CI)	*τ*	*I* ^2^
IPSS						
	Baseline	15	1580	22.04 (20.31, 23.76)	3.333	96.6%
	1–6 months	14	1250	6.09 (5.34, 6.84)	1.331	90.6%
	12 months	8	978	6.02 (3.97, 8.07)	2.923	98.4%
	24 months	3	547	7.31 (5.91, 8.72)	1.162	89.3%
	36 months	3	547	7.33 (6.59, 8.08)	0.529	65.0%
	48 months	3	547	6.17 (5.15, 7.18)	0.808	83.5%
	60 months	2	217	6.88 (6.17, 7.58)	0.000	0.0%
QoL						
	Baseline	14	1250	4.62 (4.43, 4.80)	0.327	89.2%
	1–6 months	12	1122	1.30 (1.08, 1.52)	0.368	92.1%
	12 months	6	618	1.33 (1.13, 1.52)	0.192	64.3%
	24 months	2	217	1.35 (0.86, 1.84)	0.325	84.5%
	36 months	2	217	1.39 (1.00, 1.78)	0.240	72.3%
	48 months	1	101	1.30 (1.07, 1.53)	0.000	0.0%
	60 months	1	101	1.30 (1.05, 1.55)	0.000	0.0%
*Q*_max_						
	Baseline	13	1491	8.65 (7.66, 9.64)	1.769	96.5%
	1–6 months	13	1491	19.64 (17.95, 21.34)	2.896	90.6%
	12 months	3	388	19.59 (16.86, 22.31)	2.217	85.7%
	24 months	3	547	19.16 (17.89, 20.43)	0.908	67.2%
	36 months	3	547	19.30 (17.47, 21.12)	1.366	77.8%
	48 months	2	431	17.39 (16.81, 17.97)	0.000	0.0%
	60 months	2	217	17.24 (16.18, 18.30)	0.000	0.0%
PVR						
	Baseline	13	1495	140.14 (111.77, 168.52)	50.357	97.9%
	1–6 months	12	1165	38.01 (28.84, 47.18)	15.379	97.7%
	12 months	6	864	46.33 (29.91, 62.74)	19.475	98.7%
	24 months	3	547	44.58 (42.37, 46.79)	0.000	0.0%
	36 months	3	547	36.00 (29.87, 42.13)	3.725	38.6%
	48 months	2	431	32.55 (26.51, 38.60)	3.232	23.6%
	60 months	2	217	52.95 (31.59, 74.31)	14.152	84.3%

Abbreviations: IPSS, IPSS Total Score; QoL, IPSS Qualify of Life Score.

**FIGURE 2:**
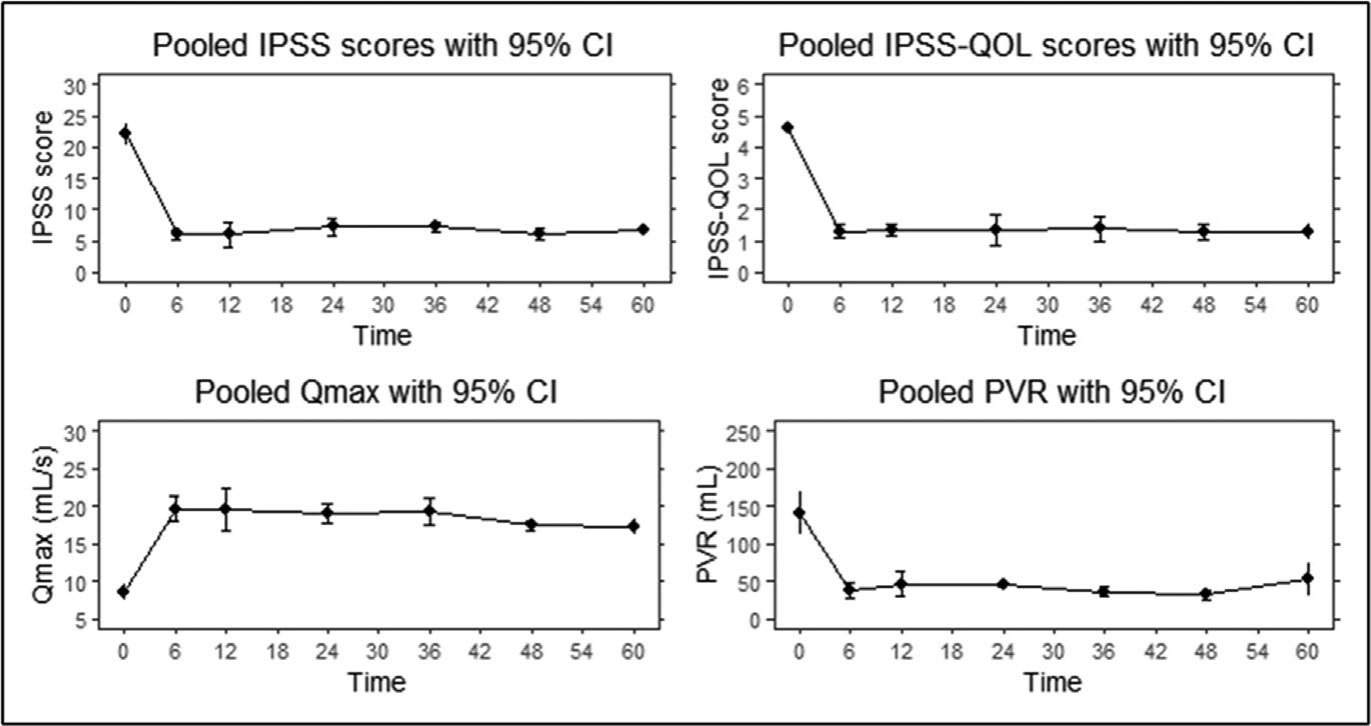
Pooled summary effect sizes with 95% CI for IPSS, QoL, *Q*_max_ and PVR over time (months).

**FIGURE 3:**
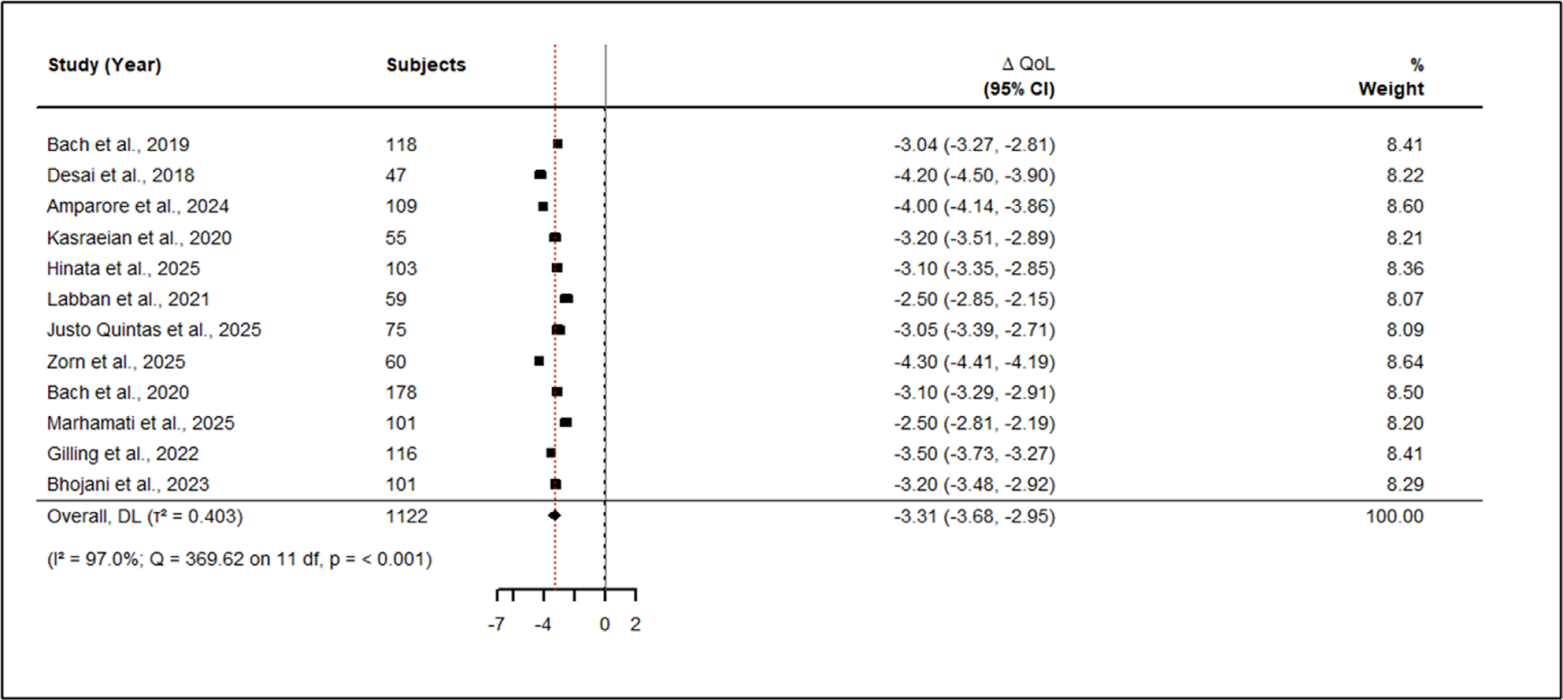
Summary effect sizes of preintervention to 1–6 months postintervention change in QoL.

*Q*_max_ improved significantly from baseline at all follow‐up periods (Figure 4). *Q*_max_ increased by 11.0 mL/s (95% CI 8.93 to 13.02, *I* ^2^ = 95.1%, *p* < 0.001) in 13 studies when followed up till 6 months and remained stable through to 36 months when reported in three studies (Figure 2). Studies reporting outcome beyond 36 months were limited (*k* < 3). PVR decreased markedly from baseline (Figure 5). Large reductions were observed when followed up till 6 months (−103.8 mL, 95% CI −139.5 to −68.0 mL, *I* ^2^ = 98.1%, *p* < 0.001), sustained at 12 months (−63.7 mL, 95% CI −75.3 to −52.1 mL, *I* ^2^ = 75.2%, *p* < 0.001), 24 months (−76.6 mL, 95% CI −85.8 to −67.3 mL, *I* ^2^ = 24.5%, *p* < 0.001) and 36 months (−83.6 mL, 95% CI −93.5 to −73.6 mL, *I* ^2^ = 28.0%, *p* < 0.001) (Table 3 and Figure 2). Between‐study heterogeneity was high in earlier periods of follow up but negligible at longer follow up, reflecting limited data rather than true homogeneity.

**FIGURE 4:**
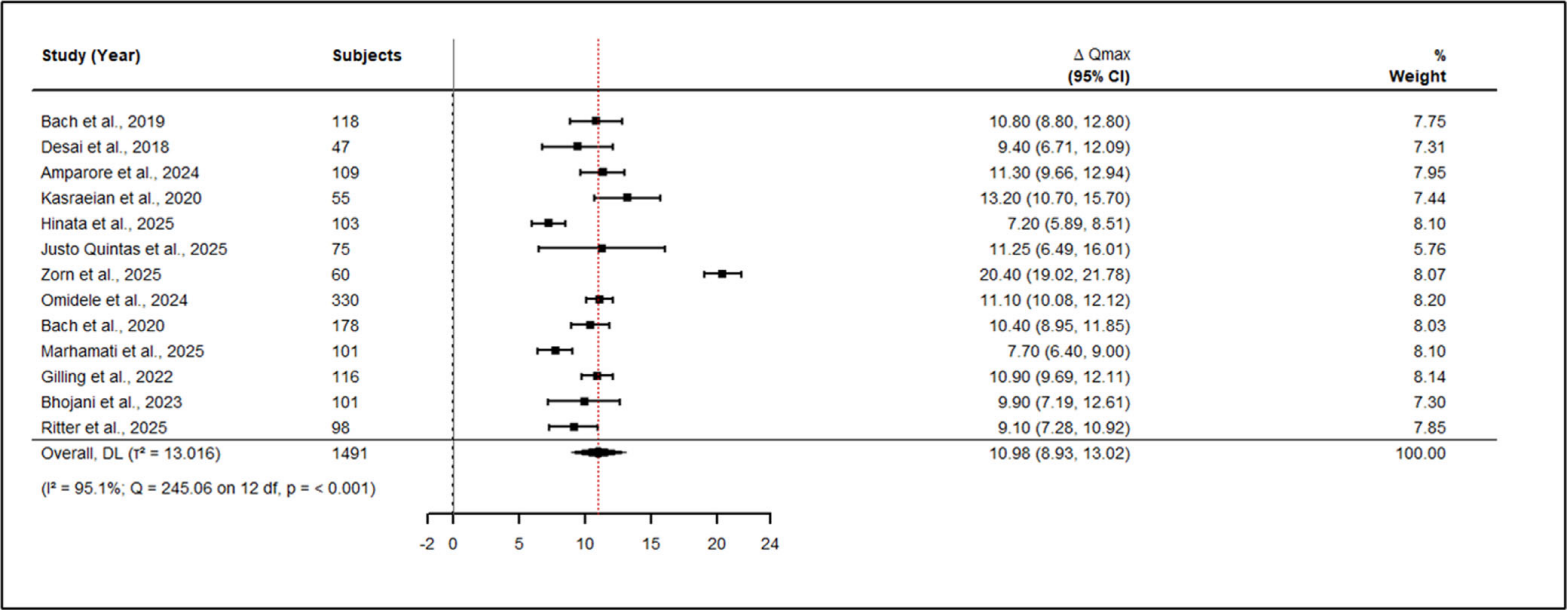
Summary effect sizes of pre‐intervention to 1–6 months postintervention change in *Q*_max_.

**FIGURE 5:**
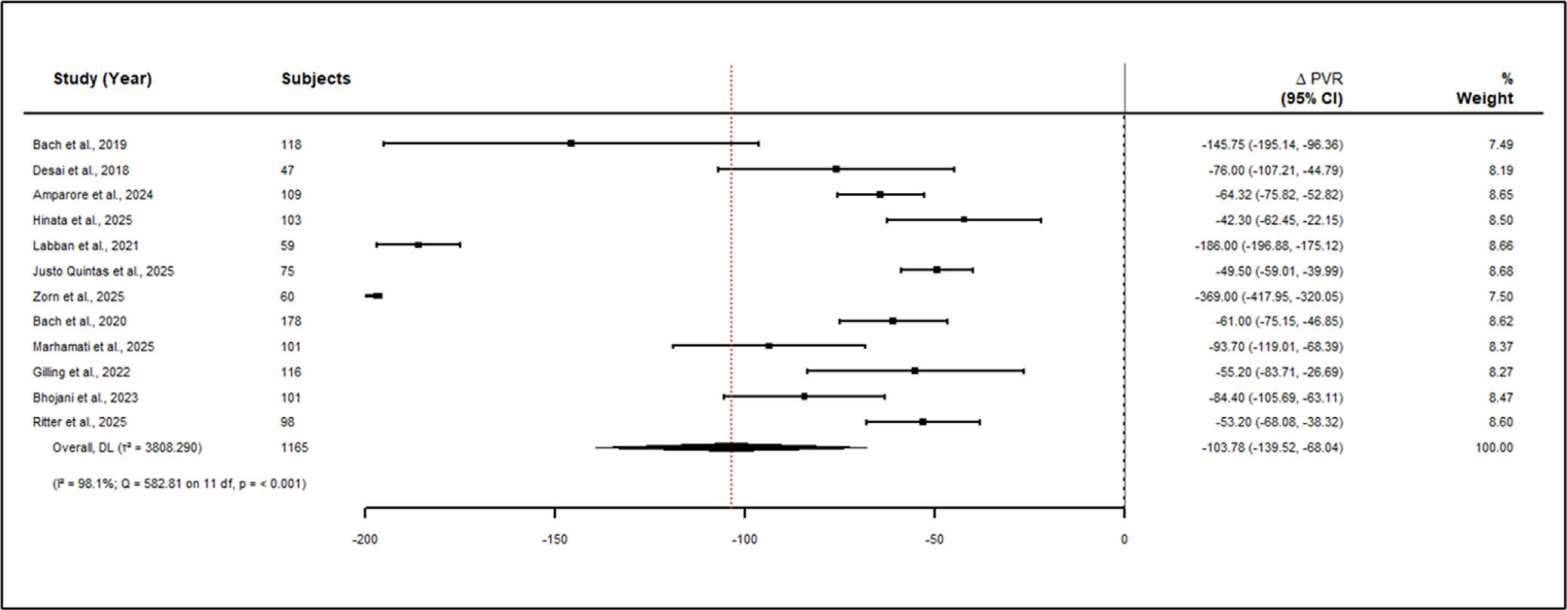
Summary effect sizes of pre‐intervention to 1–6 months postintervention change in PVR.

### 3.5 Sexual outcomes

In terms of ejaculatory and erectile function following Aquablation, studies which reported these outcomes did not necessarily use a standardised reporting system or questionnaire. Therefore, a meta‐analysis based on these measures were not included. For 13 studies that reported ejaculatory dysfunction, the pooled rate was 7.4% (95% CI 4.6%–11.8%, *I* ^2^ 75.5%). The moderate heterogeneity observed likely reflect variability in baseline function, assessment methods and outcome definitions. Twelve studies reported no observation of erectile dysfunction following Aquablation. Stress incontinence was reported in 11 studies, with a pooled rate of 1.0% (95% CI 0.5%–2%, *I* ^2^ 0%).

### 3.6 Adverse events

In the post 2020 era using focal bladder neck cautery, bleeding related complications are uncommon. The pooled rate for blood transfusion reported across all included studies was 2.5% (95% CI 1.6%–4.0%, *I* ^2^ 39.7%). A subanalysis based of haemostatic technique on blood transfusion was performed (Table 4). The lowest transfusion rate observed was observed in studies using focal bladder neck cautery (FBNC), with a pooled transfusion rate of 1.5% (95 CI: 0.7%–3.1%) across six studies (*n* = 546). Five studies (*n* = 518) employing mixed techniques demonstrated a pooled transfusion rate of 2.2% (95% CI: 1.2%–4.1%). A relatively higher blood transfusion rate was observed in two studies (*n* = 131) using traction for haemostasis (pre 2020 era), with a pooled rate of 8.4% (95% CI: 3.9%–17.3%). The bleeding complications were higher for procedures performed prior to 2020 versus the more contemporary publications. The pooled rate of return to theatre, reported in 13 studies, was 4.1% (95% CI 2.9%–5.8%, *I* ^2^ = 28.5%). Complications were reported according to the Clavien–Dindo classification.^31^ Eleven studies reported grade II complications, with a pooled rate of 11.3%. Twelve studies reported grade III and IV complications, occurring at rates of 7.5% and 0.9%, respectively.

**TABLE 4:** Random‐effects meta‐analysis of transfusion rates by haemostasis technique.

Haemostasis technique	*k*	*N*	Observed rate (95% CI)	Pooled rate (95% CI)	*τ*	*I* ^2^
FBNC	6	546	0.9% (0.3%, 2.1%)	1.5% (0.7%, 3.1%)	0.000	0.0%
Mixed	5	518	1.9% (0.9%, 3.5%)	2.2% (1.2%, 4.1%)	0.000	0.0%
Traction	2	131	8.4% (4.3%, 14.5%)	8.4% (3.9%, 17.3%)	0.315	14.7%

*Note*: Random‐effects models used DerSimonian–Laird estimation of logit‐transformed proportions, with heterogeneity summarised using *τ* and *I* ^2^.

## 4 DISCUSSION

TURP is considered the ‘reference standard’ surgical treatment option for patients with moderate‐to‐severe LUTS with prostate size between 30 and 80 cc for the last 100 years.^32^ Numerous techniques over the last 20 years have attempted to overtake TURP but ultimately lacked broad adoption due to lack of durable outcomes. Thirty years ago, laser enucleation was developed, showing good efficacy and durability. However, this has not been broadly adopted like TURP primarily due to the high learning curve and lengthy procedure times.

This systematic review and meta‐analysis demonstrate that Aquablation is safe and efficacious and is a strong alternative to traditional surgical management of BPH, with statistically significant and clinically durable improvements in all key parameters (IPSS, *Q*_max_, PVR), low risk of ejaculatory dysfunction while preserving sexual function. The efficiency and reproducibility of the procedure are made possible because the integration of imaging and robotics.

At 6 months, patients treated with Aquablation demonstrated substantial improvements in IPSS, with a 71.7% reduction (−15.80), which was sustained at 12 months (71.2%, −15.69; *k* = 8). These findings are consistent with meta‐analysis by Elterman et al.,^11^ which demonstrated significant improvements in IPSS by 16 points at 1 year across all studies. Similarly, Chen et al.^10^ also reported significant improvements in IPSS following Aquablation in their systematic review and meta‐analysis, supporting the reproducibility of symptomatic improvement across different study populations. Improvements in QoL closely paralleled symptom score reductions in our meta‐analysis, showing a 71.2% decrease in QoL bother at 1–6 months (*k* = 13), indicating a sustained patient‐perceived benefit.

From an objective functional standpoint, patients exhibited a mean increase in *Q*_max_ of 11.0 mL/s (+126.9%) at 6 months (*k* = 13), which remained stable at 36 months (*k* = 3) compared with baseline. Improvements in *Q*_max_ appeared to mirror reductions in PVR, although to a slightly lesser extent. PVR decreased by 74.1% at 1–6 months (*k* = 12) and by 59.7% at 36 months (*k* = 3). Although not all studies reported functional outcomes beyond 12 months, long‐term 5‐year follow‐up data from studies such as WATER I demonstrated continued benefit and durability, with sustained improvements in both IPSS and *Q*_max_. The high heterogeneity observed at early follow‐up likely reflects clinical differences between studies, inherent variability in uroflowmetry measurements, and interpersonal variability in urinary flow rates.

Regarding sexual function, our meta‐analysis demonstrated a 7.4% rate of ejaculatory dysfunction and no reported incidence of de novo erectile dysfunction following Aquablation.

However, reporting was inconsistent: Only eight of 13 studies assessed ejaculatory outcomes using validated questionnaires (MSHQ‐EjD), and eight of 12 studies reported erectile function outcomes using the IIEF‐15 or IIEF‐5. Among the included studies, only the WATER I trial provided data beyond 12 months, showing a minimal decline in MSHQ‐EjD scores at 5 years, which suggests durable preservation of ejaculatory function over time. The low rate of sexual side effects is likely attributable to the precise, image‐guided, and robotically executed tissue resection, allowing selective apical sparing. The characteristic “butterfly” apical cut is thought to contribute significantly to the lower rates of ejaculatory dysfunction observed with Aquablation.

Our findings are supported by recent evidence specifically evaluating sexual outcomes following Aquablation.^33^ In a systematic review of 15 studies comprising 1533 patients, Bettencourt et al. reported preservation rates of antegrade ejaculation ranging from 72.0% to 99.6%, while erectile function remained stable across the studies reviewed.^33^ Although these findings support the favourable sexual‐function profile of Aquablation, heterogeneity in outcome assessment and follow‐up duration (12 months only) remains a limitation. Further robust, long‐term data are therefore required to establish the durability of ejaculatory and erectile function preservation following Aquablation.

One of the principal safety concerns associated with Aquablation is postoperative bleeding necessitating blood transfusion or return to theatre. In our meta‐analysis, the overall rates of blood transfusion and return to theatre were 2.5% and 4.1%, respectively. However, individual studies reported considerable variability, with return to theatre and blood transfusion rates as high as 7.9%^28^ and 9.9%, respectively,^27^ in some studies, whereas no such events were reported in others.^22^, ^23^, ^24^, ^30^ These discrepancies reflect differences in haemostatic techniques, with earlier studies before 2020 relying on robust balloon catheter traction alone, without fulguration, to achieve haemostasis,^16^, ^27^ which was associated with higher rates of bleeding post‐operatively. The subsequent adoption of focal bladder neck cautery followed by standard balloon traction in later studies^18^, ^19^, ^22^, ^23^, ^25^, ^31^ significantly reduced bleeding risk, irrespective of the size of the prostate. Focal bladder neck cautery involves removal of ‘fluffy’ remnant prostatic tissue at the prostate cavity using the loop on a resectoscope followed by identification of bleeding vessels and effectively cauterising them.^10^ This approach is now regarded as the standard haemostatic technique during Aquablation and should be routinely implemented in all patients.

This systematic review is not without limitations. First, due to the relative novelty of Aquablation as a surgical treatment for benign prostatic hyperplasia, many of the included studies had relatively small patient cohort sizes, despite our inclusion criterion requiring more than 30 participants. Among the studies included, seven had fewer than 100 patients. Second, there was substantial variability in follow‐up duration and inconsistencies in reported outcome time points. Most studies reported follow up between 1 and 12 months, with only three studies reporting longer term outcomes at 48–60 months. The heterogeneity in follow up timeframe limits direct comparison of study results. Further studies with larger cohorts and longer‐term follow up are needed to provide a more reliable assessment of the durability of Aquablation. Third, there was notable heterogeneity among the included studies with respect to study design and reported outcome measures, particularly sexual outcomes, which limited uniform analysis. Finally, differences in surgical technique, the inclusion of studies reporting early institutional experiences with Aquablation, and variability in surgeon or centre procedural volume may have influenced the reported perioperative and functional outcomes.

## 5 CONCLUSION

This systematic review and meta‐analysis demonstrate that Aquablation is a safe and effective surgical treatment option for patients with BPH‐related LUTS, providing clinically durable and statistically significant improvements in urinary functional outcomes. These benefits are accompanied by favourable sexual side effects profile, with low rates of erectile and ejaculatory function. Overall, Aquablation represents an effective surgical alternative to conventional surgical treatment for BPH regardless of size.

## AUTHOR CONTRIBUTIONS

All authors were involved significantly in the makeup of this manuscript. KC lead in conceptualization, methodology, original drafting, writing of manuscript, review and editing. KC, NCG, NV researched literature and conceived the study idea alongside all listed authors in this review. KC, NCG, NV carried out data extraction. KC, NCG carried out quality assessment. KC, NCG, NV carried out statistical analysis with the guidance of MF, JN, PC, NB, JT, BC, GP, EL, DE, NV. MF, NV reviewed the manuscript and supervised throughout. KC, NV reviewed, edited the manuscript and prepared it for submission.

## CONFLICT OF INTEREST STATEMENT

There is no conflict of interest for our authors.

## Data Availability

The data that support the findings of this study are available from the corresponding author upon reasonable request.
